# Losmapimod Protected Epileptic Rats From Hippocampal Neuron Damage Through Inhibition of the MAPK Pathway

**DOI:** 10.3389/fphar.2019.00625

**Published:** 2019-06-07

**Authors:** Min Li, Lexiang Cui, Xuemin Feng, Chao Wang, Yinmeng Zhang, Lijie Wang, Ying Ding, Teng Zhao

**Affiliations:** ^1^Department of Neurology, The First Hospital of Jilin University, Changchun, China; ^2^Major in Clinical Medicine, Medical College of Nanchang University, Nanchang, China; ^3^Department of Traditional Chinese Medicine, General Hospital of FAW, Fourth Hospital of Jilin University, Changchuan, China; ^4^Department of Radiology, The First Hospital of Jilin University, Changchun, China

**Keywords:** losmapimod, mitogen-activated protein kinase, epilepsy, hippocampus, pilocarpine

## Abstract

**Objective:** This research aimed to validate the therapeutic effect of losmapimod and explore the underlying mechanism in its treatment of epilepsy.

**Methods:** A rat model of epilepsy was constructed with an injection of pilocarpine. Microarray analysis was performed to screen aberrantly expressed mRNAs and activated signaling pathways between epileptic rats and normal controls. A TdT-mediated dUTP nick-end labeling (TUNEL) assay was used to identify cell apoptosis. Hippocampal cytoarchitecture was visualized with Nissl staining. The secretion of inflammatory factors as well as the marker proteins in the mitogen-activated protein kinase (MAPK) pathway were detected by Western blot. A Morris water maze navigation test evaluated the rats’ cognitive functions.

**Results:** Activation of the MAPK signaling pathway was observed in epilepsy rats. A decrease in the MAPK phosphorylation level by application of losmapimod protected against epilepsy by reducing neuron loss. Losmapimod effectively improved memory, reduced the frequency of seizures, protected the neuron from damage, and limited the apoptosis of neurons in epilepsy rats.

**Conclusion:** The application of losmapimod could partly reverse the development of epilepsy.

## Introduction

Epilepsy is a common neurological disorder, featuring an enduring predisposition to epileptic seizures due to hyperexcitability and hypersynchrony in brain neurons (Devinsky et al., 2013; Fisher et al., 2014) and affecting approximately 50 million people approximately worldwide, with a heavy burden brought on by individual complications, medical costs, related disability, and mortality (Moshe et al., 2015; Rana and Musto, 2018). Despite the application of antiepileptic drugs (AEDs) including phenobarbital, phenytoin sodium, carbamazepine, and sodium valproate in clinical therapy in the past decade, one third of epilepsies were still refractory, providing neither relief from epileptic seizures nor modification of epileptogenesis, resulting in an urgent need for effective drugs (Tadokoro et al., 1991; Perucca et al., 2007; Loscher and Schmidt, 2011).

Mitogen-activated protein kinases (MAPKs) consist of a family of protein–serine/protein–threonine kinases followed by proline, prevalent in mammals (Pearson et al., 2001), which can be activated by tyrosine kinase activity upstream through signal transduction (Oldenhof et al., 2002). As a pivotal biochemical cascade, the MAPK signaling pathway is involved in diverse fundamental events in cells and signal transduction pathways, namely, differentiation, proliferation, apoptosis, activation of other specific regulatory factors, acute responses to hormones, and developmental alteration of organisms (Pearson et al., 2001; Bandyopadhyay et al., 2010; Gorter et al., 2014). Consequently, abnormalities in this pathway are disastrous, inducing various carcinomas such as colorectal cancer as well as neurodegenerative diseases such as Alzheimer’s disease, Parkinson’s disease, and motor neuron diseases (Corcoran et al., 2012; Gorter et al., 2014), making the MAPK pathway a target for drug development (Kim and Choi, 2010).

Activated by stress, p38 MAPKs regulate vital cellular responses such as migration, contraction, cytokine production, and death in macrophages and myocardial and endothelial cells (Marber et al., 2010; Marber et al., 2011; Denise Martin et al., 2012; DeNicola et al., 2013). In myocardial injury models, inhibition of p38 MAPK decreased the infarction size (Ma et al., 1999; Li et al., 2006; Surinkaew et al., 2013), limited postinfarction remodeling, (See et al., 2004), and attenuated atherosclerosis progression (Seeger et al., 2010). In addition, the p38 MAPK inhibitor effectively shortened the duration of seizures in epileptic rats (Yang et al., 2018). Drion et al. (2018) also reported that curcumin could promote the phosphorylation of MAPK and influence the MAPK pathway in hippocampal sections of post-SE (status epilepticus) rats. The MAPK pathway also was reported to contributed to cognitive damage in pentylenetetrazole-induced epilepsy by downregulated levels of caspase 3 (Huang et al., 2017), and inhibition of the MAPK pathway reduces antiepileptic drug resistance in refractory epileptic rats (Shao et al., 2016). The p38MAPK signaling pathway participates in drug resistance in refractory epilepsy. Inhibition of p38 MAPK also suppressed the elevation of high-sensitivity C-reactive protein (hsCRP) concentrations after percutaneous coronary intervention (PCI) (Sarov-Blat et al., 2010).

As an inhibitor of p38 MAPK α and β isoforms, losmapimod was applied for acute coronary syndrome (ACS) and chronic obstructive pulmonary disease (Lomas et al., 2012; Ostenfeld et al., 2013). In cardiovascular disease, losmapimod attenuated vascular inflammation, concentrations of circulating hsCRP and interleukin-6 (Elkhawad et al., 2012), and, subsequently, enhanced vascular function in nitric oxide-dependent or -independent patients with untreated hypercholesterolemia (Cheriyan et al., 2011). A study of losmapimod-treated inflammation in other diseases like rheumatoid arthritis and neuropathy was also reported (Yang et al., 2013). However, whether losmapimod has an effect on epilepsy remains unclear.

In this paper, we investigated the protective role of losmapimod in epileptic rat hippocampal neurons. In addition, further study revealed the participation of the MAPK signaling pathway in the progression of epilepsy. Our study might provide a novel therapeutic strategy for epilepsy.

## Methods and Materials

### Microarray Analysis

Gene expression profiling was conducted based on the GSE14763 microarray data from the GPL2896 microarray platform when rat hippocampi were subjected to pilocarpine immediately (or at least no later than 12 h) (Okamoto et al., 2010). Hippocampus tissues were collected immediately (up to 12 h) after the first spontaneous seizure (chronic). Differentially expressed genes were determined *via* the “limma” R package. Differentially expressed mRNAs in epilepsy with |log_2_FC| > 2 and *P* < 0.05 were screened.

### MRNA Processing by GSEA and STITCH

Total mRNA expression data were uploaded to GSEA v3.0 software. Gene set enrichment analysis was conducted with the KEGG (Kyoto Encyclopedia of Genes and Genomes) pathway gene set from the latest Kyoto Encyclopedia of Genes and Genomes database (http://www.genome.jp/kegg). Then, the seven most significant up/downregulation were screened out and underwent graphics processing *via* the “ggplot2” R package. In addition, the STITCH database was used as a resource to explore known and predicted interactions of chemicals and proteins, including direct (physical) and indirect (functional) associations. Default weighted enrichment statistics were adapted to process data 1,000 times with *P* < 0.05 as the significance level.

### Cell Culture and Epileptiform Activity Induction

Human hippocampal neurons (SHBIO, Shanghai, China) were divided into four groups (Ctrl, Ctrl + Los, epileptiform, and epileptiform + Los). Cells from the Ctrl group were incubated in 90% RPMI-1640 (HyClone) supplemented with 10% FBS (fetal bovine serum) at 37°C in a humidified, 5% CO_2_ atmosphere. A magnesium-free (MGF) physiological solution at pH 7.3 replaced the former medium for inducing epilepsy, containing (units in mM) 145 NaCl, 2.5 KCl, 10 *N*-2-hydroxyethylpiperazine-*N*’-2-ethanesulfonic acid (HEPES), 1 CaCl_2_, 10 glucose, and 0.001 glycine. Neurons in the epileptiform group were kept in MGF for 3 h. Neurons in the epileptiform + Los group were kept in MGF with 1 μM losmapimod for 3 h, and neurons in the Ctrl + Los group were kept in RPMI-1640 medium with 1 μM losmapimod for 3 h. After treatment, neurons were transferred to the conventional RPMI-1640 medium and maintained as previously described (Geng et al., 2018). Cells in the Ctrl group were maintained in RPMI-1640 medium under the same incubation conditions.

### Pilocarpine-Induced Status Epilepticus (SE)

Animal assays were approved by the Animal Ethics Committee of the First Hospital of Jilin University. One hundred adult male Sprague–Dawley rats aged 6–8 weeks and weighing 180–220 g were purchased from the National Institutes of Health (Bethesda, MD, USA), then randomly separated into control (30 rats) and SE (70 rats) groups. To prevent peripheral cholinergic pilocarpine effects, subcutaneous methylscopolamine (1 mg/kg, Sigma, St. Louis, MO, USA) was administered. Thirty minutes later, a pilocarpine (30 mg/kg; P6503; Sigma) intraperitoneal injection was administered to induce SE. Pilocarpine hydrochloride was given repeatedly (10 mg/kg, i.p.) every 30 min until the rats developed seizures. All rats that survived SE and presented spontaneous seizures after the silent period were considered epileptic. Rats in the control group were treated with lithium chloride and atropine sulfate as well, but the pilocarpine was replaced by normal saline. Finally, 57 SE rats were obtained, among which 45 were randomly sorted into three groups for further study. SE + Los (n = 15), SE + Pheno (n = 15), SE alone (n = 15), and control rats (n = 30) were intraperitoneally injected with losmapimod, phenobarbital, saline, and saline at 1.8 mg/kg, respectively, starting 4 h after SE and once daily for 3 days thereafter. Twenty-four hours after the last administration, three rats from each group were sacrificed to harvest their hippocampus tissues, and another 12 rats from each group were submitted to a Morris water maze (MWM) test.

### Hippocampus Extraction

SE rats were anesthetized with isoflurane (Baxter Healthcare, Compton, UK), then sacrificed to obtain their brains, from which hippocampi were dissected on ice and sliced transversally at 400 μm with a McIlwain Tissue Chopper (Mickle Laboratories, *Guildford*, UK). Thereafter, slices were transferred to 30-mm-diameter membrane inserts (Millicell, Millipore) for the following assays. The remaining tissues were stored at −80°C.

### Gene Expression Analysis

Total RNAs were isolated from tissue using TRIzol Reagent (Invitrogen, Carlsbad, CA, USA). The concentration of RNA was measured using a Qubit 2.0 Fluorometer (Invitrogen, Carlsbad, CA, USA), and cDNA from each sample was reverse transcribed using SuperScript VILO MasterMix (Life Technologies). Gene expression was detected by quantitative reverse transcription polymerase chain reaction (qRT-PCR) using SYBR Premix Ex Taq II (Takara, Dalian, China). Fold change relative expression was calculated using the ΔΔCt method with one naïve control group that served as a reference and GAPDH Ct values that served as endogenous controls for mRNA analysis. The following primers were used for the RT-qPCR analysis: GAPDH, For: 5’-TGCCACTCAGAAGACTGTGGATG-3’, Rev: 5’-GCCTGCTTCACCACCTTCTGAT-3’; NR1A, For: 5’-CGGCTCTTGGAAGATACAGC-3’, Rev: 5’-GTGAAGTGGTCGTTGGGAGT-3’; NR2A, For: 5’-GGGCGTGTTCTACATGCTG-3’, Rev: 5’-AATGTGTACCCCATGGATGCA-3’; NR2B, For: 5’-GTGAGAGCTCCTTTGCCAAC-3’, Rev: 5’-GTCAGGGTAGAGCGACTTGC-3’ NR2C, For: 5’-CTTCTGGGGGATGGAGAGAC-3’, Rev: 5’-CTGAAAGCCAGCAGGAAGTC-3’; NR2D, For: 5’-TAGTGTCAGTGCGCAGATCC-3’, Rev: 5’-ACCATGAACCAGACGTAGCC-3’; GRIA1, For: 5’-CGAGTTCTGCTACAAATCCCG-3’, Rev: 5’-TGTCCGTATGGCTTCATTGATG-3’; and GRIA2, For: 5’-CCAAGGACTCGGGAAGTAAGG-3’, Rev: 5’-CCCCCGACAAGGATGTAGAA-3’.

### TdT-Mediated dUTP Nick-End Labeling (TUNEL) Staining

Human hippocampal neurons were cultured on poly-L-lysine-coated coverslips at a density of 5 × 10^4^ cells/cm^2^, fixed in 4% paraformaldehyde for 20 min at room temperature, and washed three times in phosphate-buffered saline (PBS). Hippocampal slices were treated with 3% hydrogen peroxide, then added to the equilibration buffer. Next, the specimens were incubated with terminal deoxynucleotidyl transferase (TdT) and digoxigenin dNTPs for 60 min at 37°C followed by peroxidase-coupled anti-digoxigenin for 30 min at room temperature, then cultured in 3,3’-diaminobenzidine (DAB) substrate, counterstained with methyl green, rinsed, dehydrated, and mounted. A negative control without TdT was employed in case of nonspecific nucleotide interference or enzyme-conjugate binding. The average DAB staining intensity was considered as the apoptosis extent. Following four PBS washes, a mounting medium containing 0.5 μg/mL DAPI nuclear counterstain (Pierce, Rockford, IL, USA) was applied to the sections, and slides were coverslipped. Nine random fields were chosen by a blinded observer and used to quantify the apoptosis rate.

### Nissl Staining

The hippocampus comprises four cornu ammonis (CA) sections called CA1, CA2, CA3, and CA4. Among them, the first three are mainly filled with pyramidal cells in the stratum pyramidale (Coras and Blumcke, 2015). The cells of the CA3 section were visualized by a Nissl staining assay. Hippocampal sections (25 μm) were stained by toluidine blue (Beyotime Institute of Biotechnology, Shanghai, China) following the manufacturer’s instructions. The slides were then rinsed in distilled water, dehydrated by gradient concentrations of ethanol (70%, 80%, 90%, and 100%), cleared in xylene, and finally coverslipped with neutral balsam. The total number of cells in CA3 in each section from three nonoverlapping 400× fields was counted (Olympus Corporation, Tokyo, Japan) *via* an image analysis system (Leica Qwin Analysis software V2.8). Cells with a clear nucleus and boundary were also counted. The cross-sectional area in the hippocampus was detected by the ImageJ software (US National Institutes of Health). Nine random fields were chosen by a blinded observer and used to quantify positive cell numbers.

### Western Blot

Proteins from the hippocampal tissue were extracted with RIPA lysis buffer (Beyotime, Shanghai, China) treatment, and the concentration of proteins was measured by using a BCA (bicinchoninic acid) Protein Assay Kit (Boster Biological Technology, Wuhan, China). After extraction, the proteins were supplemented with loading buffer and boiled at 95°C for 10 min. Then, 50 μg of lysates was loaded into each well for 10% polyacrylamide gel (Boster Biological Technology) electrophoresis at 80 and 120 V. After that, wet transference was performed at 100 mV for 70 min on polyvinylidene fluoride (PVDF) membranes. Next, the membranes were blocked in 5% bovine serum albumin (BSA) at room temperature for 1 h, and primary antibodies anti-p38 (1:1,000, ab31828, Abcam, Cambridge, UK), anti-p38 (phospho T180 + Y182, 1:1,000, ab4822, Abcam), anti-β-actin (1:1,000, ab8227, Abcam), anti-IL-6 (1:1,000, ab9324, Abcam), anti-IL-1β (1:1,000, ab2105, Abcam), anti-IL-10 (1:1,000, ab34843, Abcam), anti-TNF-α (1:1,000, ab6671, Abcam), anti-CRP (1:1,000, ab50861, Abcam), anti-Bcl-2 (1:1,000, ab196495, Abcam), anti-Bax (1:1,000, ab53154, Abcam), and anti-cleaved caspase 3 (1:1,000, ab2302, Abcam) were added for incubation overnight at 4°C. Following Tris-buffered saline tween (TBST) washing for 5 min three times, secondary antibody IgG (1:1,000, ab150077, Abcam) was added at room temperature for 1-h incubation. After another washing for 5 min three times, immunoreactive proteins were detected by enhanced chemiluminescence (Thermo Fisher Scientific, Waltham, MA, USA) and analyzed by a Gel Dol EZ imager (Bio-Rad, Hercules, CA, USA). β-Actin served as a loading control. The gray value of each protein band was analyzed *via* ImageJ software. The experiments were run three times to calculate mean values.

### Morris Water Maze

Forty-eight rats (12 rats from each group) were evaluated on their ability to locate a hidden platform to escape from water using a maze tank measuring 70 cm high and 180 cm in diameter where the stages were maintained at 2 cm below the water level in four labeled quadrants. Before the test, rats were allowed to adapt to the new environment for at least 30 min. The first part of the test, place navigation, was performed once a day from 9:00 to 11:00 am four times consecutively, and a spatial probe was used on the fifth day as the second part of the test. To start the trial, rats were oriented toward the pool wall and placed into the water at different locations. They then had to swim to find the hidden stage in order to rest, and the length of time they stayed on the stage (escape latency) was recorded. Staying on the stage for no less than 2 s was recognized as the rat being well trained. If a rat failed, it would be guided to the stage by the experimenter and placed there for a certain length of time to determine a 2-min incubation period. On the fifth day, the hidden stage was removed, and rats were placed in the pool in the same way. The number of times that the rats crossed the removed stage and the duration of time spent at the location of the stage were recorded.

### Observation Index

The Racine method of epileptic seizure classification was described in a previous study (Racine, 1972). According to this standard, the incubation period of epileptic seizures in rats was recorded for 10 min. The incubation period of epileptic seizure includes the incubation period of clonic cramps and the incubation period of tetanic convulsions. The transition time from clonic cramping to tetanic convulsions was calculated.

### Statistical Analysis

GraphPad Prism 6.0 software was employed for the data analysis, expressing the results as the mean ± standard deviation (SD). Comparison of the data among all groups was analyzed by one-way ANOVA and between two groups by *t*-test. *P* < 0.05 was considered significant.

## Results

### DEGs in Epilepsy and Related MAPK Signal Pathways

**Figure 1:**
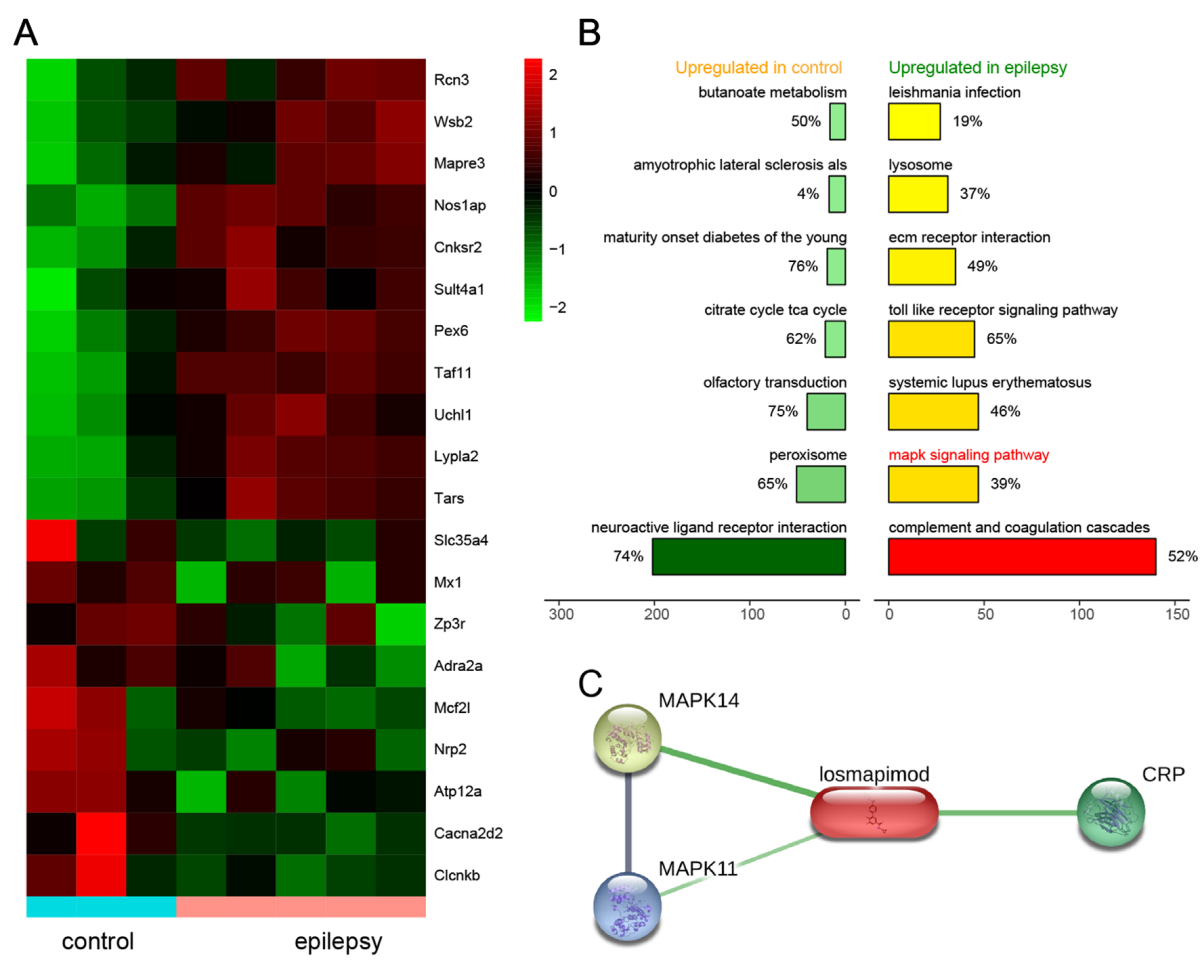
Differentially expressed genes in epilepsy and a plot of the seven most enriched KEGG pathways in epilepsy. **(A)** A heatmap shows differentially expressed genes between the healthy and epilepsy group. **(B)** A plot of the seven most enriched KEGG pathways in epilepsy. The expression fold in the epilepsy group was calculated compared with the healthy group. Pathways are ordered by normalized enrichment score (NES). **(C)** Protein–protein interactions are presented in gray and chemical–protein interactions in green; association intensity is indicated by line thickness.

Based on microarray platform GPL2896 as well as microarray data GSE14763, differentially expressed genes were screened between healthy rats (n = 3) and epileptic rats (n = 5). Several of the most upregulated and downregulated mRNAs in the epilepsy group are presented in **Figure 1** **A**. The top seven activated pathways associated with the differentially expressed genes (DEGs) are presented in **Figure 1** **B**, including the MAPK signaling pathway. In addition, the losmapimod-interacted proteins were predicted by STITCH, among which MAPK14, MAPK11, and CPB showed the strongest association with losmapimod (**Figure 1** **C**).

### The Effect of Losmapimod in Cell Apoptosis of Epileptiform Hippocampal Neurons

**Figure 2:**
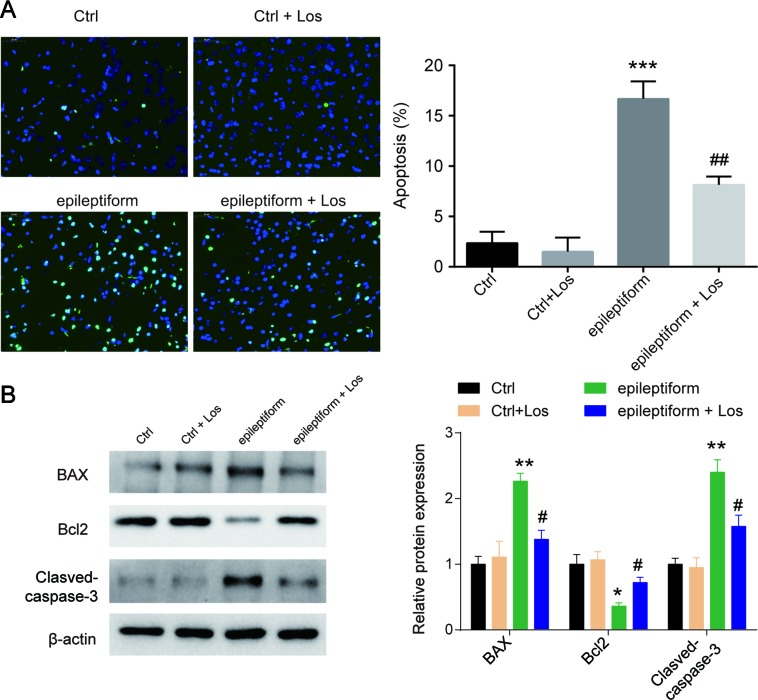
The effect of losmapimod on cell apoptosis and cell proliferation in the proto-hippocampal neurons of pilocarpine-induced epilepsy. **(A)** Cell apoptosis was detected by TUNEL assay in normal and epileptiform hippocampal neurons with or without losmapimod treatment *in vitro* (n = 3). Nine random fields were chosen by a blinded observer and used to quantify positive cell numbers. **(B)** Apoptosis-related proteins were detected by Western blot in normal and epileptiform hippocampal neurons with or without losmapimod treatment *in vitro* (n = 3). Data are all presented as the mean ± SD. *P < 0.05, **P < 0.01, ***P < 0.001 vs. Ctrl group, ^#^ *P* < 0.05, ^##^ *P* < 0.01 vs. epileptiform hippocampal neurons.

Cell apoptosis was higher in the epileptiform group than in the control groups, but losmapimod treatment rescued cell apoptosis in hippocampal neurons. The Ctrl + Los group behaved similarly as the Ctrl group (**Figure 2** **A**). To further verify this result, the expression of apoptotic proteins, such as Bax, Bcl2, and cleaved caspase 3, was also detected. Bax and cleaved caspase 3 were markedly upregulated in epileptiform hippocampal neurons, while Bcl2 was significantly downregulated. Losmapimod treatment obviously reversed the expression of Bax, Bcl2, and cleaved caspase 3 in epileptiform hippocampal neurons *in vitro* (**Figure 2** **B**). These data suggest that losmapimod treatment could reduce apoptosis in epileptiform neurons with no harm to normal cells.

### Losmapimod Affects MAPK/NF-κB-Dependent Signaling and the Expression of Inflammatory Cytokines in Epileptiform Hippocampal Neurons

**Figure 3:**
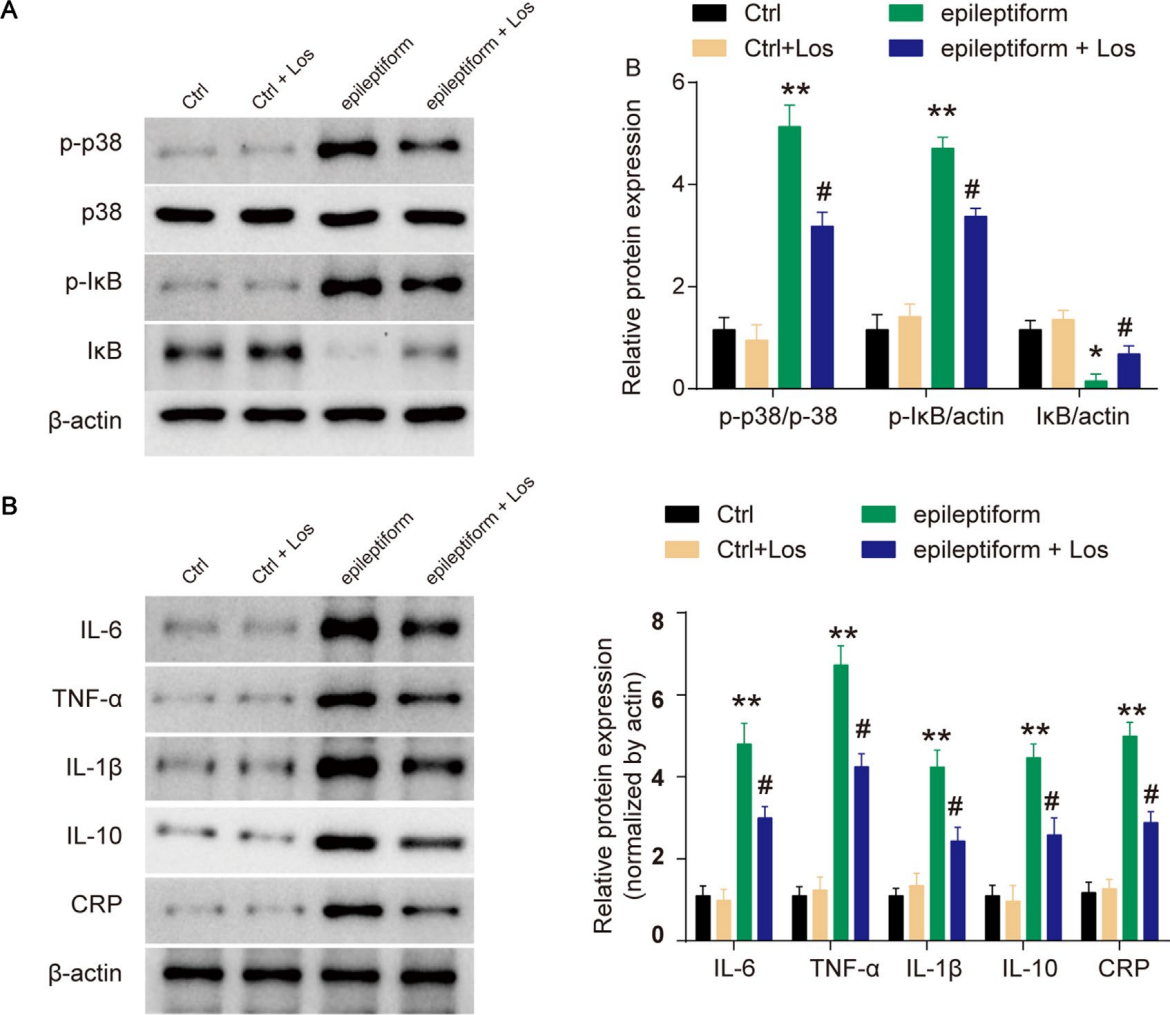
Losmapimod upregulated p-p38 MAPK and decreased the activity of inflammatory cytokines in proto-hippocampal neurons of epilepsy. **(A)** The protein expressions of p-p38, p38, p-IκB, and IκB were detected by Western blot in normal and epileptiform hippocampal neurons with or without losmapimod treatment *in vitro* (n = 3). **(B)** IL-6, TNF-α, IL-1β, IL-10, and CRP levels were detected by Western blot in normal and epileptiform hippocampal neurons with or without losmapimod treatment *in vitro* (n = 3). Each sample was measured in triplicate. The data were normalized to the internal control, β-actin. Data are all expressed as the mean ± SD. *P < 0.05, **P < 0.01 vs. Ctrl, ^#^ *P* < 0.05 vs. epileptiform hippocampal neurons.

The expression of p-p38 was obviously upregulated in epileptiform hippocampal neurons compared with control neurons (**Figure 3** **A**). Losmapimod, a selective p38-α/β MAPK inhibitor, markedly inhibited the phosphorylation of p38 in epileptiform hippocampal neurons *in vitro* (**Figure 3** **A**). In addition, the expression of p-IκB was upregulated and IκB expression was downregulated in epileptiform hippocampal neurons, and losmapimod treatment significantly reversed this change. These results suggest that MAPK/NF-κB-dependent signaling was involved in epileptiform hippocampal neurons after losmapimod treatment. Losmapimod also affected inflammatory responses in epileptiform hippocampal neurons. The levels of TNF-α, IL-1β, IL-6, IL-10, and CRP were all enhanced strikingly in epileptiform hippocampal neurons (*P* < 0.05), and losmapimod treatment suppressed these indices and reversed their trends (**Figure 3** **B**).

### Losmapimod Administration Restrained Pilocarpine-Induced Epilepsy

**Figure 4:**
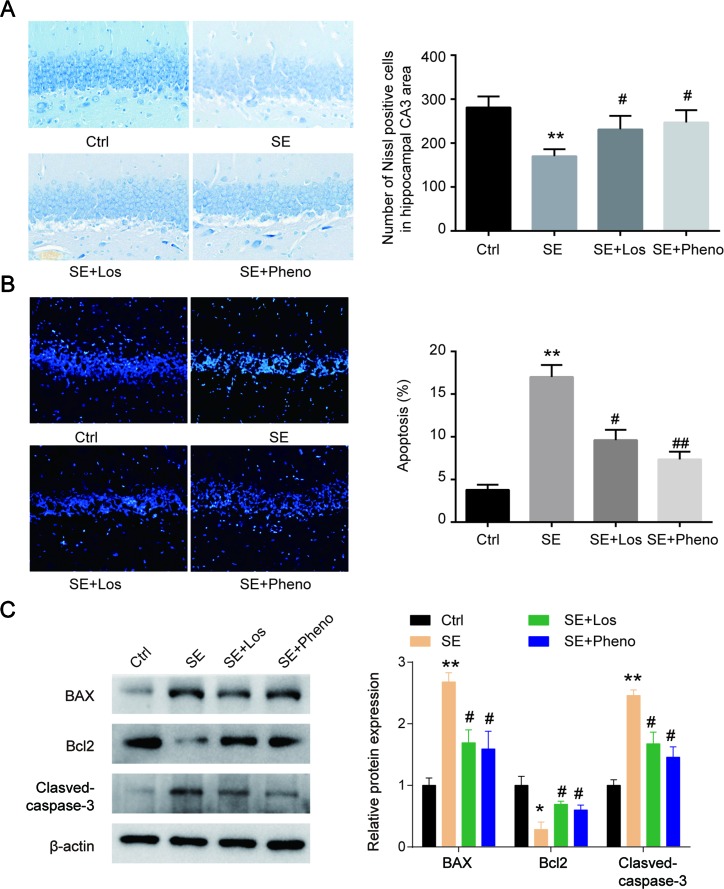
Hippocampal cytoarchitecture visualized with Nissl staining and TUNEL assays indicated cell apoptosis. **(A)** Hippocampal regions and quantitative data of the number of Nissl-positive hippocampal neurons in CA3 from rats in the four groups *in vivo* (n = 3). **(B)** TUNEL assays and quantitative data of apoptosis in hippocampal neurons from rats in the four groups *in vivo* (n = 3). Nine random fields were chosen by a blinded observer and used to quantify positive cell numbers. **(C)** Apoptosis-related proteins were detected by Western blot in hippocampal tissues in the four groups *in vivo*. Data are all presented as the mean ± SD. Scale bar = 100 mm in Figure A. *P < 0.05, **P < 0.01 vs. Ctrl, ^#^ *P* < 0.05, ^##^ *P* < 0.01 vs. SE group.

Neurons were degenerated in CA3 of pilocarpine-induced rats’ hippocampi. Following losmapimod treatment, neuronal cell loss was inhibited compared with the SE group. In addition, the results of the losmapimod-treated group was similar to that of the phenobarbital-treated group (**Figure 4** **A**). These data suggest that losmapimod treatment could rescue the loss of neurons in the CA3 region. Cell apoptosis in the pilocarpine-induced epilepsy group was higher than that in the control group, but losmapimod treatment restrained apoptosis, though the effect was not as effective as it was with phenobarbital (**Figure 4** **B**). Bax and cleaved caspase 3 were upregulated, while Bcl2 was significantly downregulated in the hippocampal tissues of the SE rats. Losmapimod treatment significantly reversed the changes in Bax, Bcl2, and cleaved caspase 3 expression in hippocampal tissues *in vivo* (**Figure 4** **C**).

**Figure 5:**
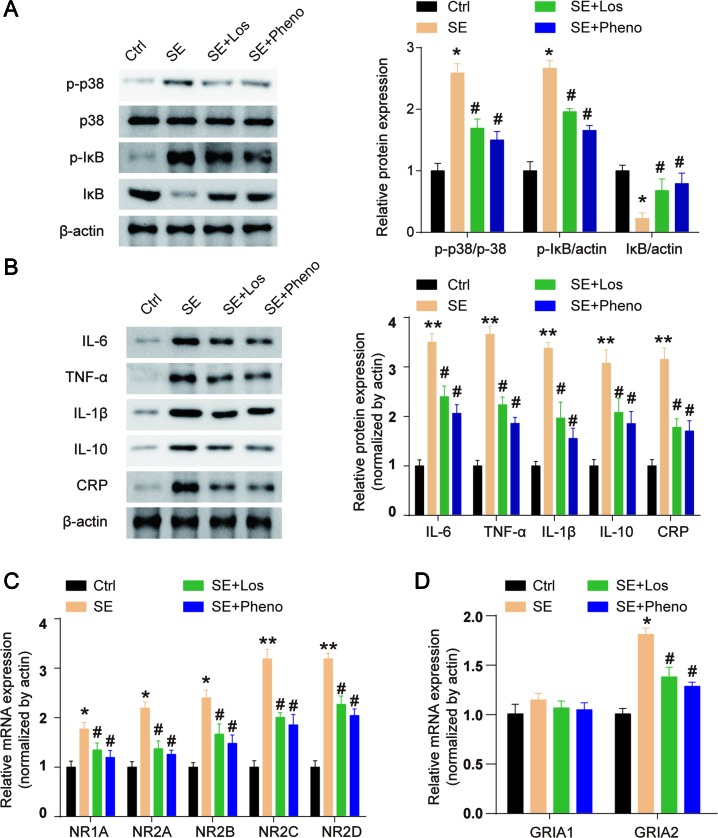
Losmapimod upregulated p-p38 MAPK and decreased the activity of inflammatory cytokines in pilocarpine-induced epilepsy. **(A)** The protein expressions of p-p38, p38, p-IκB, and IκB were detected in hippocampal tissues of SE rats *in vivo* (n = 3). **(B)** IL-6, TNF-α, IL-1β, IL-10, and CRP levels were detected in hippocampal tissues of SE rats *in vivo* (n = 3). **(C)** NR1A, NR2A, NR2B, NR2C, and NR2D mRNAs were detected in hippocampus tissues of SE rats (n = 3). **(D)** GRIA1 and GRIA2 mRNAs were detected in hippocampus tissues of SE rats (n = 3). The data were normalized to the internal control, β-actin. Each sample was measured in triplicate. Data are all presented as the mean ± SD. *P < 0.05, **P < 0.01 vs. Ctrl, ^#^ *P* < 0.05 vs. SE group.

The expression of p-p38 and p-IκB was increased, and IκB expression was decreased in the hippocampal tissues of SE rats (**Figure 5** **A**). Losmapimod treatment obviously reduced p-p38 and p-IκB expression and enhanced IκB levels *in vivo* (**Figure 5** **A**). This result reveals that losmapimod could suppress the activation of the MAPK/NF-κB signaling pathway *in vivo*. The levels of TNF-α, IL-1β, IL-6, IL-10, and CRP were all promoted remarkably in pilocarpine-induced epilepsy (*p* < 0.05), and losmapimod treatment suppressed these indexes, similar to the effect of phenobarbital (*p* < 0.05), as shown in **Figure 5** **B**.

NMDAR and AMPAR both play important roles in epilepsy. NMDA receptors (NMDARs) are hetero-oligomers composed of two NR1 subunits and one or more types of the NR2 subunit (A-D) (Wenthold et al., 2003). We found that the gene expressions of NR1A, NR2A, NR2B, NR2C, and NR2D were all upregulated in hippocampal tissues of SE rats, and losmapimod and phenobarbital treatment significantly but partly reversed these changes (**Figure 5** **C**). Transcript levels of the AMPAR GRIA2 subunit in the hippocampal tissues were markedly upregulated in SE rats, and losmapimod and phenobarbital treatment both reversed GRIA2 expression (**Figure 5** **D**). However, transcript levels of GRIA1 were not significantly different between normal rats and SE rats (**Figure 5** **D**). Losmapimod treatment could reverse NMDAR and AMPAR expressions, which indicated that losmapimod may protect epilepsy through regulating synaptic protein distribution.

### Losmapimod Ameliorated Cognitive Deficits in Pilocarpine-Induced Epilepsy

**Figure 6:**
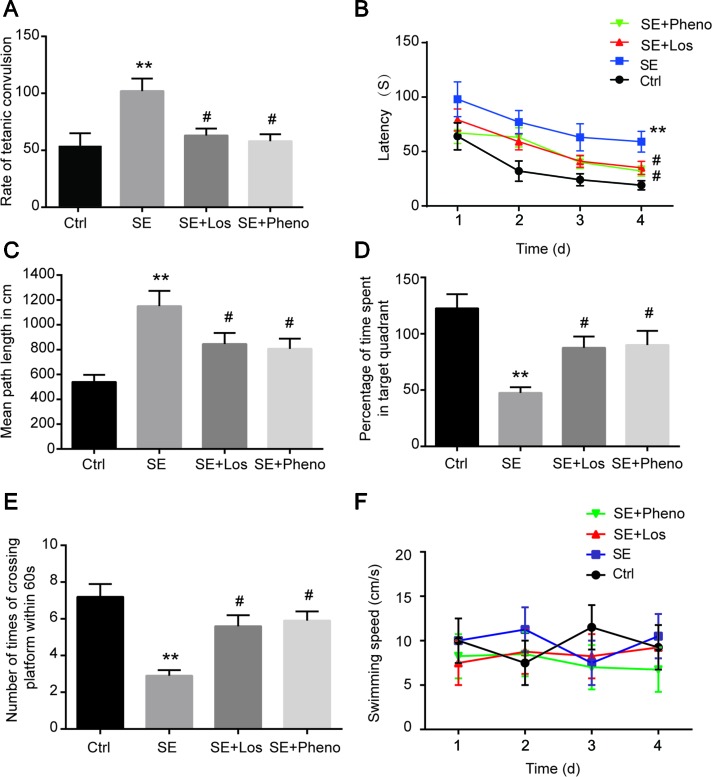
Profiles of behavioral testing by Morris water maze. **(A)** The rate of tetanic convulsions, **(B)** escape latency, **(C)** mean path length, **(D)** percentage of time spent in the target quadrant, **(E)** number of times the platform was crossed, and **(F)** swimming speed in each group (n = 12). **P < 0.01 vs. Ctrl, ^#^ *P* < 0.05 vs. SE group. Ctrl: control, SE: pilocarpine-induced status epilepsy, SE + Los: epilepsy treated by losmapimod, SE + Pheno: epilepsy treated by phenobarbital.

The incubation period and transition time of epileptic seizures were increased in the SE group compared with the control group. In addition, treatment with losmapimod increased the incubation period and transition time of epileptic seizures, which was the same effect seen with phenobarbital-treated SE rats (**Table 1**). To evaluate the cognitive effects of losmapimod, a Morris water maze test was performed. **Figure 6** **A** indicates that losmapimod reversed the changes caused by epilepsy in the rate of tetanic convulsions. Pretreatment for 4 days with losmapimod reduced the mean escape latency (*P* < 0.05), which was the same with phenobarbital in treating epilepsy, as illustrated in **Figure 6** **B**. Likewise, **Figure 6** **C** also indicates an evident elevation of mean path length in the epilepsy group compared to the control group (*P* < 0.05), which was obviously prevented after pretreatment with losmapimod and phenobarbital (*P* < 0.05). On the other hand, significant decreases in the time that the rats stayed in the target quadrant and in the times that the rats crossed the former stage were observed in the epilepsy group compared to the control group (*P* < 0.05), as shown in **Figure 6** **D** and **E**, respectively, indicating the spatial memory effect of losmapimod in SE rats. However, supplementation with losmapimod reversed the trend in pilocarpine-induced epilepsy rats during the probe trial (*P* < 0.05). For swimming speed, no statistical significance was discovered among the groups, as shown in **Figure 6** **F**. Taken together, these findings strongly support the hypothesis that losmapimod may reverse the dysfunction of learning and memory in epileptic rats.

**Table 1:** The effect of losmapimod on the seizure latency of epileptic rats.

Group	Incubation period of clonic cramp (s)	Transition time (s)	Incubation period of tetanic convulsion
Control	N.A.	N.A.	N.A.
SE	63.7±18.1	27.1±12.6	92.1±31.7
Los	92.8±36.6	40.1±19.9	126.9±48.6
Pheno	94.1±43.6	38.3±21.7	137.1±50.2

## Discussion

Recurrent seizure discharges, memory abnormality, and conscious impairment are some of the various syndromes in clinical epilepsy. The two most important complications of epileptic seizures are learning and memory deficits. Previous investigations have shown that the frequency of discharge in the electroencephalogram (EEG) during awake and sleep was more than 10%, resulting in cognitive impairment in adult patients (Lv et al., 2013). Indeed, the longer the duration of the epilepsy, the more that cognition was impaired (Jokeit and Ebner, 1999). In this article, we found that this cognition impairment can be reversed by the intraperitoneal injection of losmapimod and associate this phenomenon with neuronal apoptosis.

The MAPK signaling pathway was chosen to act as a link between epilepsy and apoptosis. In KEGG enrichment analysis, our results showed that the MAPK signaling path was activated in epileptic samples. MAPK was reported to be an important regulator of synaptic excitability, exerting an influence on cognitive impairment and epilepsy in animal models as well as human disease (Pernice et al., 2016). Xi et al. (2007) discovered the upregulation of MAPK in intractable epilepsy. It was demonstrated that the cognitive deficits in pentylenetetrazole (PTZ)-induced epilepsy was associated with the abnormal expression of p38 MAPK (Huang et al., 2017). Huang et al. (2017) found that the expression of p-p38 was upregulated in a PTZ-induced epileptic model, which was similar to our study. Lopes et al. (2012) found the overexpression of p-p38 in the hippocampus and cortex of pilocarpine-induced epileptic rats, whereas decreased p38 activity was observed after kainic acid treatment in another study (Mielke et al., 1999), implying that p38 modifies the epileptogenic network.

Usually, cell apoptosis might be the terminal step of many signaling pathways, and thus, the relationship among apoptosis, epilepsy, and cognitive deficits has become a frequently researched topic over the last few decades (Mendez-Armenta et al., 2014; Teocchi and D’Souza-Li, 2016; Wang et al., 2016). For example, cognitive impairments and neuronal apoptosis were detected in epileptic patients and animal models (Jafarian et al., 2015; Zhang et al., 2017), suggesting their close link to epilepsy. In our research, we also observed an increase in apoptosis in epileptic rats. However, losmapimod and phenobarbital both decreased neuronal cell loss, which might be an underlying reason for the losmapimod treatment of epilepsy.

Furthermore, Alam et al. reported that the behavioral effect of p38 was mediated by a reduction of cytokine production (Alam, 2015). The increased secretion of inflammatory factors exacerbated epileptic seizures (Das et al., 2012). Losmapimod, as an inhibitor of p38 MAPK, was expected to be effective in treating cytokine-dependent inflammatory diseases (Watz et al., 2014). In our research, we found that the protein expression of pro-inflammatory factors, including TNF-α, IL-1β, IL-6, and IL-10, was higher in pilocarpine-induced epilepsy. In addition, the treatment of losmapimod reversed these results. The activation of NF-κB-dependent signaling deteriorated cognitive dysfunction and promoted neuronal apoptosis (Zhang et al., 2013). In our research, NF-κB-dependent signaling was activated in SE rats, and losmapimod treatment may alleviate SE pilocarpine-induced epilepsy by inhibiting the activation of MAPK/NF-κB-dependent signaling.

It was reported that the serum level of CRP was upregulated in epileptic patients and could be a biomarker for epileptic patients (Talaat et al., 2015; Zhou et al., 2018). CRP is an important molecule related to the acute phase of the inflammatory process, the main effect of which is the modulation of several cascades related to immune system activation (Gewurz et al., 1995). IL-6 is the principal inducer of CRP gene expression, while IL-1, TNF-α, and activated complement act synergistically with IL-6 to enhance its effect (Ganapathi et al., 1991; Szalai et al., 2000). In our study, the expression of CRP was upregulated in the hippocampal tissue of SE rats, and losmapimod treatment partially reversed this change.

Ionotropic glutamate receptors (iGluRs) mediate the synaptic and metabolic actions of glutamate. These iGluRs are classified into the α-amino-3-hydroxy-5-methyl-4-isoxazole propionic acid (AMPA)-type, kainate-type, and *N*-methyl-d-aspartate (NMDA)-type functional receptor families. NMDAR activation plays a pivotal role in the pathophysiology of several neurological disorders, such as hypoxia-ischemia, trauma, and epilepsy (Olney, 1990). In addition to NMDAR, AMPAR also plays an important role in epilepsy (Gross, 2019). In our study, NMDAR and AMPAR gene expressions were found to be upregulated in the hippocampal tissue of epilepsy rats. Losmapimod treatment could reverse NMDAR and AMPAR (speciﬁcally GRIA2) expressions, which indicated that losmapimod may protect against epilepsy through regulating synaptic protein distribution.

It is worth noting that several inadequacies in our findings were not neglectable. Since p38 MAPK participates in various cellular processes including cell growth, apoptosis, and cytokine production (Peluso et al., 2017), as an inhibitor, losmapimod should also have an effect on multiple systems in addition to the abovementioned. For example, hippocampal tissue contains not only neurons but also microglial cells and astrocytes that can also produce cytokines. The internal relationship between cell and cell and between cell and losmapimod is therefore a new target for future research. In this study, only male rats were employed, which limits our conclusions as there are sex differences in seizure types and symptoms.

In conclusion, losmapimod could protect hippocampal neurons in epileptic rats by reducing hippocampal neuronal cell loss and inflammation secretion, resulting in relieving the symptoms of epilepsy in rats.

## Ethics Statement

Animal assays were approved by the Animal Ethics Committee of the First Hospital of Jilin University.

## Author Contributions

ML, LC, XF, and CW provided substantial contribution to the conception and design of the work. CW, YZ, and LW performed analysis and interpretation of the data. ML drafted the manuscript. YD and TZ revised the work critically. Final approval of the work was done by all authors.

## Funding

This work was supported by the UCB foundation project of epilepsy research fund of the Chinese Antiepileptic Association (2019030).

## Conflict of Interest Statement

The authors declare that the research was conducted in the absence of any commercial or financial relationships that could be construed as a potential conflict of interest.
